# Gene Ontology density estimation and discourse analysis for automatic GeneRiF extraction

**DOI:** 10.1186/1471-2105-9-S3-S9

**Published:** 2008-04-11

**Authors:** Julien Gobeill, Imad Tbahriti, Frédéric Ehrler, Anaïs Mottaz, Anne-Lise Veuthey, Patrick Ruch

**Affiliations:** 1 University and Hospitals of Geneva, Geneva, Switzerland; 2 Swiss-Prot Research Group, Swiss Institute of Bioinformatics, Geneva, Switzerland

## Abstract

**Background:**

This paper describes and evaluates a sentence selection engine that extracts a GeneRiF (Gene Reference into Functions) as defined in ENTREZ-Gene based on a MEDLINE record. Inputs for this task include both a gene and a pointer to a MEDLINE reference. In the suggested approach we merge two independent sentence extraction strategies. The first proposed strategy (LASt) uses argumentative features, inspired by discourse-analysis models. The second extraction scheme (GOEx) uses an automatic text categorizer to estimate the density of Gene Ontology categories in every sentence; thus providing a full ranking of all possible candidate GeneRiFs. A combination of the two approaches is proposed, which also aims at reducing the size of the selected segment by filtering out non-content bearing rhetorical phrases.

**Results:**

Based on the TREC-2003 Genomics collection for GeneRiF identification, the LASt extraction strategy is already competitive (52.78%). When used in a combined approach, the extraction task clearly shows improvement, achieving a Dice score of over 57% (+10%).

**Conclusions:**

Argumentative representation levels and conceptual density estimation using Gene Ontology contents appear complementary for functional annotation in proteomics.

## Introduction

As an increasing amount of information becomes available in the form of electronic documents, the increasing need for intelligent text processing makes shallow text understanding methods such as the Information Extraction (IE) particularly useful. Until now, IE has been strictly defined by DARPA's MUC (Message Understanding Conference) program [1], as a task involving the extraction of specific, well-defined types of information from natural language texts in restricted domains, with the specific objective of filling predefined templates and databases. Examples of such classical information extraction tasks are given by the BioCreative named-entity recognition task or by the Joint workshop on Natural Language Processing in Biomedical Applications (JNLPBA) shared task [2]. The TREC-2003 Genomics Track and the BioCreative I passage retrieval task propose an extension of IE tasks to identify entities, such as functional descriptors, which are less strictly defined than gene and gene products. The 2003 Genomics Track suggested extracting gene functions as defined in the LocusLink database (now ENTREZ-Gene). In this repository, records (called locus, which refer to a gene or a protein) are provided with a short fragment of text to explain their biological function together with a link to the corresponding scientific article. These so-called Gene Reference Into Functions (GeneRiFs) are usually short extracts taken from MEDLINE articles. Generalizing the use of textual passages for the three axes of the Gene Ontology (GO), i.e. not only to identify functions but also to identify biological processes and cellular components, the BioCreative task 2.1 aimed at evaluating text mining tools tailored to extract short passages. The idea is to support existing or predicted GO annotation. For such tasks, advances retrieval engines are sufficient to achieve top-precision in the range of 80% or higher. Thus, mean reciprocal rank measures reaching 87.18% for retrieving passages supporting protein-protein interactions has been reported for BioCreative II [3]!

The remainder of the paper is organized as follows: first, an overview of the state of the art. Then, a description the different methods and their combinations, as well as the metrics defined for the task. Finally, reports on results and conclusion.

## Background and applications

Historically, seminal studies dedicated to the selection of textual fragments were done for automatic summarization purposes [4], but recently, due to developments in life sciences, more attention has been focused on sentence filtering, in particular to extract textual evidences of functional descriptions in life sciences [5].

### Summarization

In automatic summarization, the sentence is the most common type of text-span used: partly because computational linguistic models have historically focused on syntax, i.e. on intra-sentence relationships and, partly because cross-sentences dependencies are far more difficult to process Thus, by choosing sentences as generation units, many co-reference issues [6] are partially avoided. So that, it seems simpler and more effective to view the summarization problem as a sentence extraction problem. [4] distinguish between two summary types: generic and query-driven. This distinction is useful relative to our information extraction task, since we see the task as a direct application of question-answering applied to functional proteomics. Indeed, the study can be regarded as an attempt to answer typical questions as formulated by Swiss-Prot expert curators. In our approach, we combine a discourse-level parser, the argumentative classifier, which automatically categorizes sentences in a MEDLINE abstract into a predefined set of argumentative structures, together with a passage density estimator, driven by Gene Ontology categories.

For automatic summarization, feature selection and weighting, often based on term frequency and inverse document frequency factors (tf.idf) have been reported. Conclusions reached are however not always consistent [7] with respect to tf.idf. Among other interesting features, both sentence location as well as sentence length seem important [8]. In addition, these studies rely on lists of frequent phrases and keywords, computed on the summarization domain. Finally, to extract important sentences from documents, documents' titles and uppercase words such as named-entities are reported to be good predictors. Of particular interest for our approach, [9] define a large list of manually weighted triggers (using both words and expressions such as *we argued, in this article, the paper is an attempt to*, etc.) to automatically structure scientific articles into seven argumentative classes, namely: BACKGROUND, TOPIC, RELATED WORK, PURPOSE, METHOD, RESULT, and CONCLUSION.

### A life sciences literature perspective on Information Extraction

To date and as with gene and gene products functions, descriptions of most of the biological knowledge cannot be found in databanks, but only in the form of scientific summaries and articles [10]. Although some visionary authors [11] already identified the stake in the 90's, making use of these textual contents represents a major milestone towards building models of the various interactions between biological entities and for complex biological systems in general. For example, sentence filtering for protein interactions was previously described in [12]. In the same vein, functional annotation of proteins [13] and protein interactions, which were respectively the subject of BioCreative I and II, are tasks demanding information extraction at the level of short passages. In these studies, sentence filtering is viewed as a prerequisite step towards deeper understanding of texts. A similar design has been proposed by the TREC Genomics track in 2006, which investigated passage retrieval in full-text articles [14]. As for discourse-analysis and its role in text mining applications for molecular biology, it has been used for keyword extractions [15][16], information extraction [17], paradigm shift [18], related-article search [19], and automatic query expansion (blind feed-back) in MEDLINE ad hoc search tasks [20].

### TREC 2003 benchmarks

To provide a general view of the problems underlying the generation of the most appropriate GeneRiF during the TREC-2003 Genomics Track [21], a simple example is provided in Table 1. In this table we can see the locus (“ABCA1”) and the MEDLINE record identifier (“PMID - 12804586”). Under the label “TI”, we find the article's title and under “AB” its abstract, from which the GeneRiF is extracted. A preliminary study [21] showed that around 95% of the GeneRiF snippets were extracted from the title or from the abstract of the corresponding scientific paper. Moreover, from this set, 45% were a direct cut and paste from either the title or the abstract (Table 1 is such an example) while another 25% contained significant portions of the title or abstract. A set of 5000 GeneRiF-abstract pairs has been collected for tuning our system before evaluating it on the TREC benchmark.

**Table 1:** Example of an ENTREZ-Gene record and the corresponding GeneRiF (italic added)

**Input**

Locus - ABCA1: ATP-binding cassette, sub-family A (ABC1), member 1

MEDLINE record - PMID - 12804586 TI - Dynamic regulation of alternative ATP-binding cassette transporter A1 transcripts. AB - (…) The longest (class 1) transcripts were abundant in adult brain and fetal tissues. Class 2 transcripts predominated in most other tissues. The shortest (class 3) transcripts were present mainly in adult liver and lung. To study the biochemical significance of changes in transcript distribution, two cell models were compared. In primary human fibroblasts, upregulation of mRNA levels by oxysterols and retinoic acid increased the relative proportion of class 2 transcript compared to class 1. Phorbol ester stimulated human macrophage-derived THP-1 cells increased the abundance of class 1 transcripts relative to class 2. In both cell lines class 3 transcript levels were minimal and unchanged. It is shown here for the first time that the *regulation of ABCA1 mRNA levels exploits the use of alternative transcription start sites*.

**Output**

GeneRiF: *regulation of ABC A1 mRNA levels exploits the use of alternative transcription start sites*

**Figure 1:**
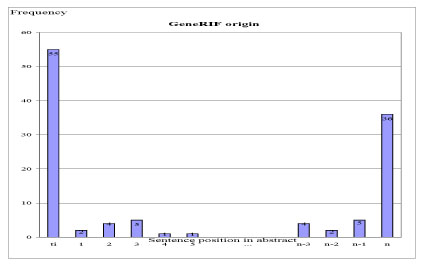
GeneRiF distribution in titles and (“ti”) and in abstracts from the 1^st^ to the *n^th^* sentence

In the TREC evaluation data, we analyzed the sentence location distribution used to produce the GeneRiF. In this case, we considered the title (see Figure 1, the first column labelled “ti”) and the abstract's sentence sequence. For the evaluation, we rely on the 139 official TREC data. From this data set, 55 were mainly extracted from the article's title, as depicted in Figure 1. The second most frequent source of GeneRiF was the abstract's last sentence (see the last column in Figure 1, following the label “n”), showing the source of 36 GeneRiFs. Between these two extreme positions, the GeneRiF location distribution is rather flat.

## Methods

As for automatic abstracting, evaluating sentence classifiers is difficult. First, establishing a consensus benchmark is clearly a more complex task. Second, it is less universally defined, as compared to other automatic text classification tasks such as spelling correction, document routing or in information retrieval systems evaluation.

### Metrics

In general, for each input text the data mining techniques yield a ranked list of candidates. Thus, sentence filtering like information extraction and text categorization may be formally evaluated by recall and precision measures. However, we must recognize that it is hard to obtain complete agreement regarding the appropriate measure that should be used for sentence comparison. It has been argued [22] that in evaluating a binary classification system, one should use effectiveness measures based on estimates of class membership rather than measures based on rankings. Elsewhere, a precision oriented-metric such as 11-point average precision has been suggested [23]. In the TREC-2003 genomics evaluation campaign, a third type of measure was used to evaluate information extraction: the Dice coefficient is shown in Equation 1. In this formula, the numerator indicates the number of common words between the candidate sentence (*X*) and the expected GeneRiF (*Y*), while the denominator represents the total number of words in the GeneRiF and in the candidate. Thus, this similarity coefficient measures the lexical overlap between a candidate and the corresponding correct GeneRiF.

$$Dice\;=\;\frac{2∥X\;\cap \;Y∥}{∥X\;+\;Y∥}$$

More precisely, four Dice coefficients variants were suggested, and all were found to be highly correlated-N-grams metrics were tested but no evidence of improvement compared to word-based distance measure was observed. In our experiments the Dice metrics given in Eq. 1 is used. This measure assumes that a binary decision was made prior to computing the Dice distance: a unique candidate GeneRiF must be selected.

### Common pre- and post-processing strategies

We started designing the task as a ranking task. But it is worth observing that in real settings, the user should first formulate an information request related to a particular protein or gene, and involving categories such as molecular functions, body or cellular locations, pathological functions, species, drugs, and tissues. In our experiments, we assume that from every document a GeneRiFs can be extracted, while in real settings, database curators need first to search and select a set of relevant documents using a retrieval/question-answering engine – For example, using the EAGLi search interface: . So in the design of the task, the system is fed with an abstract, which a priori contain a GeneRiF. As defined by the TREC Genomics protocol, we also hypothesized that GeneRiFs are sentences or significant sentence fragments. Such a statement is however questionable since some examples in the GeneRiF test data support opposite observations: GeneRiFsare sometimes the synthesis of more than one sentence. For sentence splitting, we developed a robust tool based on manually crafted regular expressions. The tool can detect sentence boundaries with more than 97% precision on MEDLINE abstracts, and was deemed competitive with more elaborate methods [24]. In order to avoid applying our classifiers on erroneously segmented sentences, segments with less than 20 characters were simply removed from the list of candidate sentences.

Each method ranks the candidate sentences separately. From these two rankings, our aim is to identify a confidence estimator on each ranking. When both methods disagree on the top-ranked sentence, a final decision is needed. This last step transformed the two ranking tools into a binary classifier, thus finally deciding whether a candidate sentence is a GeneRiF or not. The selected sentence, which is unique in each {locus, abstract} pair, is post-processed by a syntactic module, in an ultimate attempt to eliminate non content-bearing phrases from the selected sentence.

This sentence reduction step, also called trimming or compression, uses a part-of-speech tagger [25] and a standard list of 369 stopwords (e.g., *so*, *therefore*, *however*, *then*, etc.) together with a set of stop phrases (e.g., *in contrast to other studies*, *in this paper*, etc.). When these stop phrases occurred they are removed from the beginning of the selected GeneRiF candidate. Part-of-speech information is used to augment the list of stopwords, thus any adverb (e.g. *finally*, *surprisingly*, etc.) located at the beginning of a sentence are removed. In the same manner, this procedure removes non-content bearing introductory syntagms when they are located at the beginning of the sentence: any fragment of text containing a verb and ending with *that*, as in *we show that*, *the paper provides the first evidence that*, were deleted. Stopword and stop phrase removal steps are applied sequentially, but we arbitrarily limited the length of the deleted segment at a maximum of 60 characters. Moreover, sentence trimming is blocked when clauses contain gene and protein names (GPN). The GPN tagger is based on a very simple heuristic: any non-recognized English token is considered as a GPN. We use the UMLS SPECIALIST Lexicon and a frequency list of English words (total of more than 400,000 items) to separate between known and unknown words.

### Latent Argumentative Structuring

The first classifier is called LASt for Latent Argumentative Structuring, so called because we assume that it can reveal the underlying/latent/hidden rhetorical structure of the article. The tool started ranking abstract sentences as to their argumentative classes. The discourse model defines four classes: PURPOSE, METHODS, RESULTS and CONCLUSION. These classes were chosen because in scientific literature they have been found to be fairly stable [26][27] and they are also recommended by ANSI/ISO guidelines for professionals. We obtained 19,555 explicitly structured abstracts from MEDLINE in order to train our Latent Argumentative Structuring learner – this set does not contain the MEDLINE records used during the evaluation. A conjunctive query was used to combine the four following strings: “PURPOSE:” “METHODS:” “RESULTS:,” “CONCLUSION:”. From the original set, we retained 12,000 abstracts (an example is given in Table 2) used for training our LASt system, and 1,200 were used for fine-tuning and evaluating the tool, following removal of explicit argumentative markers.

**Table 2:** Example of an explicitly structured abstract in MEDLINE. The 4-class argumentation model, supporting our experiments can have minor variations in abstracts as illustrated with the INTRODUCTION marker in this explicitly structured abstracts. Explicitely structured abstracts in MEDLINE account for less than 2% of all abstracts.

**INTRODUCTION:** Chromophobe renal cell carcinoma (CCRC) comprises 5% of neoplasms of renal tubular epithelium. CCRC may have a slightly better prognosis than clear cell carcinoma, but outcome data are limited. **PURPOSE:** In this study, we analyzed 250 renal cell carcinomas to a) determine frequency of CCRC at our Hospital and b) analyze clinical and pathologic features of CCRCs. **METHODS:** A total of 250 renal carcinomas were analyzed between March 1990 and March 1999. Tumors were classified according to well-established histologic criteria to determine stage of disease; the system proposed by Robson was used. **RESULTS:** Of 250 renal cell carcinomas analyzed, 36 were classified as chromophobe renal cell carcinoma, representing 14% of the group studied. The tumors had an average diameter of 14 cm. Robson staging was possible in all cases, and 10 patients were stage 1) 11 stage II; 10 stage III, and five stage IV. The average follow-up period was 4 years and 18 (53%) patients were alive without disease. **CONCLUSION:** The highly favorable pathologic stage (RI-RII, 58%) and the fact that the majority of patients were alive and disease-free suggested a more favorable prognosis for this type of renal cell carcinoma.

#### Features and heuristics

Our system relies on four Bayesian classifiers [28], one binary classifier per argumentative class. Each binary classifier combined three types of features: words, word bigrams and trigrams. The log of the class frequency represented the weight of each feature, but for every category, DF thresholding [29] is applied so that rare features are not selected. The class estimate provided by each binary classifier is used to attribute the final class (an example is shown in Tables 2 and 3): for each sentence the classifier with the highest score assigns the argumentative category. We also investigated the sentence position's impact on the classification effectiveness through assigning a relative position to each sentence, see [30] for a comprehensive evaluation and description of the argumentative classifier.

**Table 3:** The classification results for the abstract shown in Table 2 (explicit argumentative labels are removed before classification). For each row, the attributed class is followed by the score for the class, followed by the extracted text segment. In this example, one of RESULTS sentences (in bold) is misclassified as METHODS, while he INTRODUCTION sentence has been classified as PURPOSE.

CONCLUSION (00160116) The highly favorable pathologic stage (RI-RII, 58%) and the fact that the majority of patients were alive and disease-free suggested a more favorable prognosis for this type of renal cell carcinoma.
METHODS (00160119) Tumors were classified according to well-established histologic criteria to determine stage of disease; the system proposed by Robson was used.
**METHODS (00162303)** Of 250 renal cell carcinomas analyzed, 36 were classified as chromophobe renal cell carcinoma, representing 14% of the group studied.
PURPOSE (00156456) In this study, we analyzed 250 renal cell carcinomas to a) determine frequency of CCRC at our Hospital and b) analyze clinical and pathologic features of CCRCs.
PURPOSE (00167817) Chromophobe renal cell carcinoma (CCRC) comprises 5% of neoplasms of renal tubular epithelium. CCRC may have a slightly better prognosis than clear cell carcinoma, but outcome data are limited.
RESULTS (00155338) Robson staging was possible in all cases, and 10 patients were stage 1) 11 stage II; 10 stage III, and five stage IV.

Table 4 indicates the confusion matrices between the four classes, with and without the use of relative position heuristics. When the sentence position is not taken into account, 80.65% of PURPOSE sentences are correctly classified, while 16% are mis-classified as CONCLUSION, and 3.23% as RESULTS. On the other hand, when the sentence position is taken into account, 93.55% of PURPOSE sentences are correctly classified. The data depicted in this table demonstrates that position can be useful for separating between the PURPOSE and CONCLUSION classes. However, the percentages of correctly classified sentences in the METHODS or RESULTS classes do not vary when the sentence position is taken into account.

**Table 4:** Confusion matrices for argumentative classification: the first column indeictes the expected category, while the first line provides the measured classification.

	Without sentence positions			
	PURPOSE	METHODS	RESULTS	CONCLUS.

PURPOSE	80.65 %	0 %	3.23 %	16 %
METHODS	8 %	78 %	8 %	6 %
RESULTS	18.58 %	5.31 %	52.21 %	23.89 %
CONCLUS.	18.18 %	0 %	2.27 %	79.55 %
	With sentence positions			

	PURPOSE	METHODS	RESULTS	CONCLUS.

PURPOSE	93.35 %	0 %	3.23 %	3 %
METHODS	3 %	78 %	8 %	6 %
RESULTS	12.43 %	5.31 %	52.21 %	13.01 %
CONCLUS.	2.27 %	0 %	2.27 %	95.45 %

#### Argumentation and GeneRiF

In preliminary experiment we tried to establish a relation between the GeneRiFs and the argumentative sections. We selected two sets of 1000 GeneRiFs from our training data and submitted them to the argumentative classifier. Both set A and B are random sets, but for B we impose that the extract describing the GeneRiF must be found in the abstract (as exemplified in Table 1). We want to verify that the argumentative distribution of GeneRiFs originating from abstracts is similar to the distribution of GeneRiFs originating from both titles and abstracts. Results of the argumentative classification are given in Table 5 for these two sets. These proportions indicate that GeneRiFs are mainly classified as PURPOSE and CONCLUSION sentences (respectively 41% and 55% in Set A). The significance of these observations is accentuated for GeneRiFs originating from the abstract (see Set B in Table 5) but the trend is stable. In this case, two thirds of the GeneRiFs originate from the CONCLUSION, and around a quarter from the PURPOSE section. Together, these two argumentative classes concentrate between 88% (Set B) and 96% (Set A) for the GeneRiFs in LocusLink (now ENTREZ-Gene). Fortunately, and as shown in Table 4, the discriminative power of the argumentative classifier is more effective for these two classes than for the RESULTS and METHODS classes.

**Table 5:** Class distribution in 1000 GeneRiFs after argumentative classification. Sets A and B are samples of GeneRiFs as in LocusLink, but Set B contains only GeneRiFs originating from the abstract.

	Set A (%)	Set B (%)
PURPOSE	41	22
METHODS	2	4
RESULTS	2	8
CONCLUSION	55	66

Based on these findings, the preferred sentence ranking order for GeneRiF extraction should be: CONCLUSION, PURPOSE, RESULTS, METHODS. So we expect that argumentation parsing should top-select a sentence classified as CONCLUSION. However, selecting the best CONCLUSION sentence is not sufficient (such a strategy exhibits a Dice performance of 35.2%), due to the fact that 45% of GeneRiFs in the TREC evaluation set were strictly cut and paste from the article's title. Clearly, our argumentation-based method needs to take the title into account. To do so, we simply compute the Dice distance between each candidate and the title, so that among sentences classified as CONCLUSION and PURPOSES, those lexically similar to the title would move to the top of the list. In complement, a negative filter is also used: sentences without GPNs are simply discounted. Finally, to select between the title and the best-ranked sentence from the abstract, the Dice score is again used. If the sentence score is above a given threshold, then the sentence is selected, otherwise the title is returned. From our GeneRiF training data, the best threshold is 0.5. On the test set, this threshold results in selecting 14 sentences from the abstract and 125 from the title, out of a total of 139 queries (see Table 6).

**Table 6:** Sample distribution of the most frequent GO terms in Swiss-Prot.

GO ID	Proportion (%)	Cumul. (%)	Term
0005634	3.41	3.41	nucleus
0007165	3.19	6.60	signal transduction
0005737	2.75	9.36	cytoplasm
0005887	2.58	11.9	integral to plasma membrane
0005886	1.65	13.6	plasma membrane
0003700	1.48	15.0	transcription factor activity
0016021	1.48	16.5	integral to membrane
0005515	1.04	17.6	protein binding
0006412	0.88	18.5	protein biosynthesis
0006810	0.82	19.3	transport

### Gene Ontology-driven passage selection

The second module capitalizes on the automatic assignment of GO categories to each candidate sentence. We hypothesize that sentences having a high density of GO categories are more likely to serve as GeneRiFs.

Stemming is applied to get a more general feature representation space. In parallel, for each sentence in the abstract, a vector is constructed. The title of the abstracts is normalized as other sentences to provide a candidate vector. The vector space contains a dimension for each feature of the Gene Ontology, including external resources terminological resources. In particular, Swiss-Prot keywords improved the effectiveness of the categorizer. To rank the candidate vectors we compute a vector-space distance between these candidates and the reference vector. The resulting ranking expresses a lexical similarity between the annotation provided by LocusLink curators and sentences of the abstract and title. Feature weighting is based on *tf* (term frequency), *idf* (inverse document frequency), and pivoted normalization factors applied on the Gene Ontology. The idea is that frequent lexical features in the GO controlled vocabulary (such as *receptor, nucleotid, transfer, regul*…) must be downweighted. A distribution of the most frequent GO codes is given in Table 6. The prior distribution is computed based on Swiss-Prot data as provided in the context of the BioCreative initiative for the three axes of the Gene Ontology.

The original tuning of the tool is based on experiments conducted for the BioCreative tasks 2.1 [5], where the system achieved very competitive results both for functional annotation and for passage selection – see [31] for a comparative presentation. In [5], we showed that the categorization status value (CSV) assigned by the categorizer provided an effective estimate to assess the intrinsic quality of the predicted association. In particular, we showed that we can transform our top performing recall-oriented categorizer into a competitive precision-oriented system, just by setting a threshold on the CSV, what resulted in a precision close to 80%. In the same vein, we assume here that the CSV can be directly used to estimate the density of Gene Ontology-related features.

Today the Gene Ontology categorizer [32] has significantly improved its classification power. While reports are under preparation, the GO Categorizer is already available for testing via two different interfaces: 1) a predictive model, which emphasizes knowledge discovery based on machine learning, and 2) a data-poor browsing mode, which emphasizes lexical similarities between an input document and the GO descriptors. The first demonstrator accepts a PMID or a short text as input (), while the second accepts multiple MEDLINE citations ranked according to a user query (). The predictive model currently achieves top-precision of 48.93% for a recall of 66.56% after twenty categories. The data-poor model achieves a top precision of 29.8% but is more adapted to density estimation than the predictive model; therefore it is used in the experiments reported in this paper. These results should be contrasted with the GO inter-curator agreement, which is in the range of 39%-43% [33].

For estimating the GO conceptual density, we simply sum up the categorization status values assigned to the top-N categories; with *N* = 2 determined by direct search. Table 7 provides an example of density estimation for two sentences extracted from the abstract in Table 1.

**Table 7:** Two sentences extracted from the abstract in Table 1 are assigned a ranked set of N Gene Ontology descriptors (*N* = 2). Each {sentence;category} association pair is provided with a categorization status value (CSV), which directly expresses a similarity between the sentence and the Gene Ontology. The final density is computed by simply summing up the top-N CSV (*N* = 2).

Sentences	Predicted GO categories	CSV	Density
Class 2 transcripts predominated in most other tissues	rna primary transcript binding	1028	2056
	35s primary transcript processing	1028	

regulation of ABCA1 mRNA levels exploits the use of alternative transcription start sites	transcription	9397	12819
	regulation	3422	

### Fusion of extraction strategies

This last step attempts to combine our two extraction schemes. To achieve this goal, we used the following rules (decision boundaries are computed on the GeneRiF training set):

• Agreement - if the sentence selected by LASt is also chosen by the GOEx module, then we keep it;

• Disagreement - if both ranker disagree on the top item, then we look at the density estimate returned by GOEx;

– if this estimate is above an empirical threshold and if the candidate sentence is assigned a PURPOSE/CONCLUSION/Title category by the LASt classifier, then the candidate sentence provided by GOEx is selected,

– otherwise the LASt candidate sentence is returned.

Finally, once a unique candidate GeneRiF is selected and if this segment does not come from the title, then the sentence is processed by the reduction procedure (see Common pre- and post-processing strategies). The output segment is used for comparison to the correct GeneRiF provided by LocusLink's annotators, as explained in the next section. We have tried to apply the sentence reduction filter, either after of before the combination, without observing any change regarding the final output.

## Results and discussion

Table 8 depicts the overall performance measure using the Dice coefficient (last column). The table's middle columns show how the proposed GeneRiF may have originated from the article's title or from an abstract sentence. Our baseline approach was very simple. For each of the 139 queries (composed of a locus and a MEDLINE article), we returned the article's title. Such a naive selection procedure achieved a relatively high performance of 50.47%, due to the fact that 45% of GeneRiFs were extracted from the article's title. On the other hand, if for each query we had an oracle that always selected the title or the sentence achieving the highest Dice score, we could obtain a performance of 70.96%, one that represents a theoretical upper bound for our experiments. In this optimal run, we had to extract 59 titles and 80 sentences from the abstract. We could not however obtain a better performance level due to the fact that LocusLink's annotators may have used words that did not appear in the article's title or in the abstract. Moreover, correct GeneRiFs may paraphrase a sentence or the article's title, revealing the same gene function with different words or expressions. Finally, we must keep in mind that GeneRiFs can be rewritten using more than one sentence. In this case, the human annotator chooses to combine different segments, taken from various sentences or hypothetically from the full-text of the article.

**Table 8:** Performance of each basic strategy and their combination. The top combination (57.29%) achieves 80.7% of the theoretical upper bound (70.96%).

	Origin of the proposed GeneRiF		Dice (%)
	Title	Abstract	

Baseline	139	0	50.47
LASt	125	14	51.98
LASt & shortening	125	14	52.78
GOEx	103	36	52.36
Combination	108	31	56.14
Combination & shortening	108	31	57.29

As shown in Table 8, the LASt extraction approach produced an overall performance of 51.98%, and in this case, 125 GeneRiFs came from the article's title and 14 from the article's abstract. After sentence shortening, the system achieves a Dice score of 52.78% (ranked 3 out of 15 participants in the official TREC evaluation). Our second extraction scheme (run labelled GOEx) performed at near similar levels (52.28%). However, in this case, it was seen that a greater number of proposed GeneRiFs came from the abstract (36 vs. 14 in the LASt scheme). The last two rows of Table 8 indicate the performance of our combined approach (56.14%), clearly showing better overall results than those for each extraction scheme run separately. When we apply the sentence trimming procedure, the Dice score increased slightly (57.29% vs. 56.14%). When analyzing the origin of each proposed GeneRiF in this combined approach, we could see that 108 come from the title and 31 from the abstract.

While these results reveal attractive performance levels when compared to other runs in the TREC-2003 genomic evaluation campaign [21], several teams were faced with the same extraction problem yet suggested other interesting approaches. For example, [34], ranked second at TREC (Dice = 53%) suggested a scheme that selected between the article's title and the last sentence of the article's abstract (as shown in Figure 1, 91 out of the 139 GeneRiFs were extracted from either the title or the abstract's last sentence). These authors suggested basing this selection on a Naive Bayes [35] machine learning approach. The relevant variables were the verbs, MeSH terms and the genes, all weighted by *tf.idf*, as well as a Boolean value representing the presence of the target gene in the abstract. Although we were not able to reproduce their results based on their TREC report, [36] report a Dice score close to 57%, using similar classifiers, but trained on the sentence position in the abstract. [37] report on slightly weaker results using text-derived rather than Gene Ontology-derived features, which confirms that Gene Ontology features capture most information needed for functional annotation. Another interesting approach proposed by [38] separates the articles, abstracts and titles into sentences in order to combine their various characteristics, such as the number of words, number of figures and number of uppercase letters. The first model applied a linear combination on a set of characteristics so as to extract the best candidate sentence, whereas the second model was based on the predicate calculus, using another set of characteristics.

Our results not only confirm that argumentation plays an important role to drive the extraction of functional descriptions in life sciences' texts, but also show how complementary information can be directly extracted from the Gene Ontology controlled-vocabulary using a generic text categorization engine. Further, while our categorization framework outperforms other methods regarding recall [39], precision [5], and *F — score*[40], it appears also useful to guide feature selection and to estimate prediction confidence various annotation tasks beyond category assignment, in particular for sentence selection and passage retrieval, as needed for GeneRiF extraction. With a top-precision approaching now 50%, future improvements should foster this trend. Interestingly, while sentence extraction is normally seen as a preliminary step toward assigning Gene Ontology descriptors, we observe here that such a controlled-vocabulary can be used a priori to guide the sentence selection process, from which more accurate category assignment could be expected !

Since the latent argumentative structure does not fully correlate with positional information in MEDLINE abstracts, our experiments clearly suggest that argumentation can provide contents, which are more discriminating than positional information. In addition, to strict functional annotation, as defined by the Gene Ontology along its three axes (molecular functions, subcellular location and biological processes), GeneRiFs also may describe pathological functions, drug-related information and tissue-specificity. Potentially GeneRiFs can cover most of the scope of functional molecular biology. Therefore we could expect that by estimating the density of other semantic categories, such as pathological functions (e.g. drugs or diseases using Medical Subject Headings), could significantly help extracting passages supporting protein-diseases associations, in particular for passages related to mutated proteins [41].

Furthermore, full-text articles are expected to convey more knowledge than a short abstract, but in practice, several large-scale text mining tasks do not seem to benefit from the use of full-text documents [42][5]. Indeed, together with bringing signals, full-texts also brings massive amounts of noise. By separating between novelty-related facts [18] (e.g. results and conclusions) and trivial facts (e.g. background, methods…) or between established (e.g. background) vs. putative facts (e.g. purposes and conclusion), we hope that argumentative filtering could help *reading* the mass of published articles in biomedical sciences.

## Conclusion

This paper focuses on the extraction of gene function sentences (so-called GeneRiF) from a MEDLINE record given a gene name, as proposed in the TREC Genomics Track in 2003 [21]. Because almost half of the human-provided GeneRiFs were simply cut and paste from the title, we designed a method to rank sentences according to two independent criteria: 1) discourse analysis criteria; 2) Gene Ontology conceptual density. The first method considers that, apart from the title, the best GeneRiF candidate should appear in the article's conclusion or purpose sections. The second extraction approach is based on a generic categorization framework, which is designed to estimate the density of Gene Ontology concepts in the selected sentences. Each extraction strategy uses rank-based methods and operates on the same basic unit: the sentences and/or the article's title. The combination is fully based on empirical heuristics derived from a relatively small tuning data set, avoiding overfitting phenomena.

The argumentative filtering yielded effective results during the TREC-2003 challenge [21]. Combining this approach with the Gene Ontology-driven ranking module, which takes advantage of the available protein annotation, improves the lexical overlap - measured by Dice metrics - by about 10% compared to the baseline. The system achieves more than 80% of the corresponding theoretical upper baseline. In conclusion, the methods used in these experiments provide a general view of the gene function extraction task within the framework of TREC Genomics evaluation campaigns, as well as in previous or more recent BioCreative initiatives [3]. As it is known for summarization, these methods clearly show that a wide variety of non-overlapping feature sets should be considered when performing such information extraction tasks. Relatively poorly investigated by researchers in text mining, both terminology-driven density estimation and discourse-level classification look particularly promising.

## Competing interests

The authors declare that they have no competing interests.

## Authors' contributions

• Design of the experiments : P Ruch and A-L Veuthey

• Development of the GOEx categorizer : P Ruch

• Parametrization of the GOEx's data driven model, user interfaces and web services: J Gobeill

• Task-specific tuning of GOEx: F Ehrler

• Evaluation and refinement of GOEx: A Mottaz

• Development of LASt: P Ruch

• Evaluation of LASt, design of user interfaces and web services: I Tbahriti
